# Ground Dwelling Ants as Surrogates for Establishing Conservation Priorities in the Australian Wet Tropics

**DOI:** 10.1673/031.009.1201

**Published:** 2009-04-10

**Authors:** Sze Huei Yek, Stephen E Willliams, Chris J. Burwell, Simon K.A. Robson, Ross H. Crozier

**Affiliations:** ^1^ School of Marine and Tropical Biology, James Cook University, Townsville, 4811, Australia; ^2^ Centre for Tropical Biodiversity and Climate Change, School of Marine and Tropical Biology, James Cook University, Townsville, 4811, Australia; ^3^ Entomology Section, Queensland Museum, South Brisbane, 4101, Australia

**Keywords:** altitudinal gradient, species richness, complementarity, conservation assessments, evolutionary history

## Abstract

This study aims to identify a set of areas with high biodiversity value over a small spatial scale within the Australian Wet Tropics. We identified sites of high biodiversity value across an altitudinal gradient of ground dwelling ant communities using three measures of biodiversity. The three measures considered were estimated species richness, complementarity between sites and evolutionary history. The latter measure was derived using the systematic nomenclature of the ants to infer a surrogate phylogeny. The goal of conservation assessments could then be achieved by choosing the most diverse site combinations. This approach was found to be valuable for identifying the most diverse site combinations across an altitudinal gradient that could ensure the preservation of terrestrial ground dwelling invertebrates in the Australian Wet Tropics.

## Introduction

**Figure 1.**
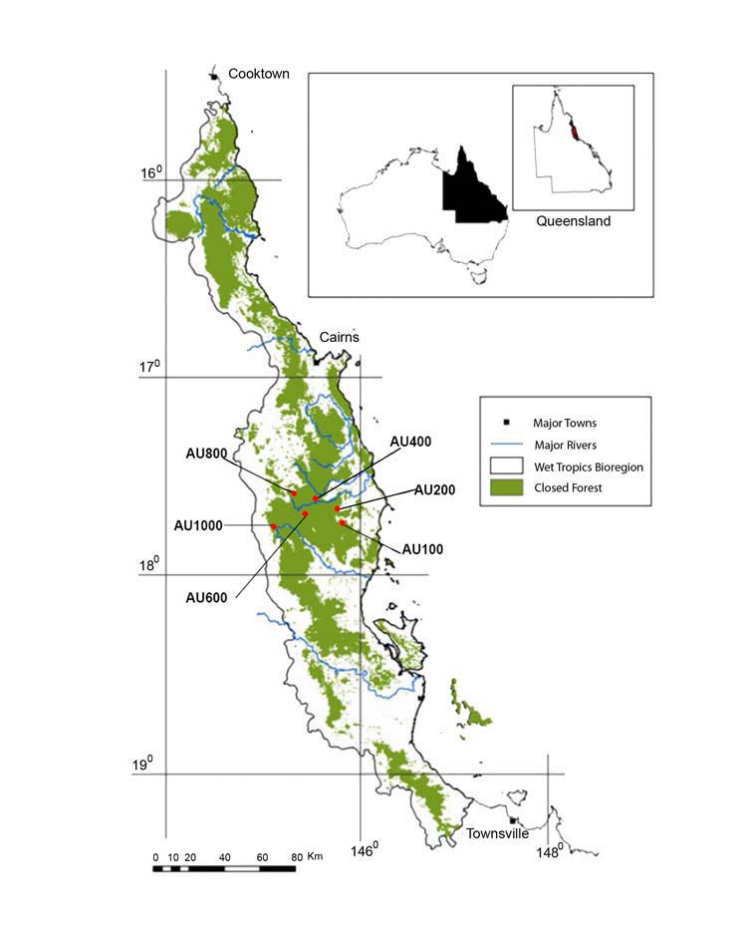
Geographical distributions of study sites in the Australian Wet Tropics

The Australian Wet Tropics is the remains of the once extensive rainforests that covered much of northern Australia (Figure 1) (Singh 1982). This region contains high levels of diversity and endemism (Wiltshire 1986; Frith 1992). It was designated a World Heritage Site in 1988 as recognition of its high biological diversity and conservation value. As this region is already protected from short-term anthropogenic disturbance, its greatest long-term threat is likely to be from global warming (see Hilbert et al. 2001; Walsh et al. 2004). It is particularly vulnerable to global warming because the terrain is dominated by mountain ranges giving extremes of altitude from sea level to around 1600 meters (Hilbert et al. 2001) with the majority of its endemic species restricted to the cooler uplands (Nix and Swizer 1991). Temperature increases due to global warming are predicted to lead to dramatic decline of highland habitat, causing widespread extinctions of the endemics (Williams et al. 2003). Given the severity of the predicted effects of global warming on the Australian Wet Tropics, there is a pressing need to make informed decisions about conservation and management priorities for this region.

In this study we chose ground dwelling ants as surrogate taxa in an attempt to establish conservation priorities for the Australian Wet Tropics. This choice is because ground dwelling ants have numerous attributes that make them ideal candidates for surrogate taxa. These attributes include high diversity (Agosti et al. 2000), relatively well resolved taxonomy (Lawton et al. 1998), ease of collection, colonies relatively stationary, and important ecological functions (Hoffmann and Andersen 2003). The knowledge of ground dwelling ant diversity in an area can provide a great deal of useful information for conservation planning (Agosti et al. 2000). Ants have been reported to correlate with the presence of other organisms and to indicate the overall health of an ecosystem (Andersen et al. 2004). Most ant species live in relatively stationary colonies, in contrast to flighted insects, hence they can be re-sampled repeatedly over time using the same method, providing reliable baseline data that can be used for long-term monitoring of environmental changes (Kaspari and Majer 2000).

Systematic conservation planning seeks to identify areas with high biodiversity and conservation value. The criteria used for identification of areas with conservation importance should be based on the persistence likelihood of species at different sites (Weitzman 1992). As an example, if two sites are equally biodiverse, conservation priority should be placed on the site that can be preserved better into the future. Therefore, besides consideration of biodiversity patterns, the ecological and evolutionary distinctiveness that maintain and generate species should also be taken into account (Cowling and Pressey 2001). The common indices for biodiversity assessments such as estimated species richness, and complementarity reveal the biodiversity pattern of an area but lack information containing evolutionary distinctiveness. Therefore it has been suggested that biodiversity assessment should take evolutionary distinctiveness into account (Vane-Wright et al. 1991; Faith 1992; Crozier 1992) as reviewed by Crozier (1997). All measures of evolutionary distinctiveness, such as evolutionary history (May 1994; Nee and May 1997), assess the biodiversity content of an area based on how much of the encompassing phylogeny of organisms is preserved (Crozier et al. 2005). Hence, the general application of this method to biodiversity assessment is limited to the small proportion of taxa that have been placed in a phylogeny. However, a large molecular study has shown how this approach can be used to prioritize sites within the Cape Flora (Forest 2007) and a study of Madagascan ants (Smith et al. 2005) shows that at least DNA barcoding data can be swiftly gathered. Crozier et al. (2005) provided proof of concept for the idea that, because systematists generally try to make nomenclature follow phylogeny, one can use systematic nomenclature to yield a surrogate phylogeny. The aim in this study is to assess the performances of three diversity indices (species richness, sites complementarity and evolutionary history) of ground dwelling ants in establishing conservation priorities across an altitudinal gradient in the Australian Wet Tropics.

## Materials and Methods

### Ground dwelling ants sampling

Ground dwelling ant sampling was carried out across an altitudinal transect in one of the most important biodiversity hotspots for the Australian Wet Tropics. This region ranges from the coastal lowlands south of Cairns up to the Atherton Tablelands, and on to the highest part of the region, the Bellenden Kerr/Bartle Frere mountain ranges (Williams et al. 2003). Six elevations (100, 200, 400, 600, 800, 1000 m) (Figure 1, Appendix 1) were selected along an altitudinal transect from this region. Sampling was carried out twice, once during November 2004 and again in February 2005. Ground dwelling ants were sampled with pitfall traps constructed from 120 ml specimen jars (4.5 cm diameter), covered by a 17-cm × 11-cm plastic container as shelter against rain. Each jar was partially filled with 100% high-grade ethanol as a killing and preservation agent. Traps were embedded in the ground with the lip of the trap flush with the ground surface. At each altitudinal transect, three sites were sampled with five pitfall traps per site, totaling up to 15 traps for an altitude (Appendix 2). At each replicate site along the altitudinal transect, traps were 1 m apart and perpendicular to the altitudinal transect. The traps were retrieved after 5 days in the sampling sites. Ant specimens were separated from other arthropods, mounted and labeled. Ants were identified to genus level following Shattuck (1999) and to species/morphospecies level by CJB and SHY; representative specimens are lodged with the Queensland Museum, Brisbane and James Cook University School of Marine and Tropical Biology, Townsville.

### Estimating species richness

The diversity of ground dwelling ants for each altitude was estimated with species accumulation curves created from computations using Estimates (Colwell 2005). In choosing richness estimators, Chao diversity estimators are generally expected to perform well in inventories of hyperdiverse arthropod groups (Gotelli and Colwell 2001). The Chao 2 richness estimator which is based on incidence data was therefore used for the estimation of species richness:

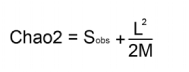

where S_*obs*_ is the observed species richness, *L* is the number of species that occur in only one sample, and *M* is the number of species that occur in only two samples (Chao 1987).

Species richness, not the numerical structure of the ant community was of interest, so the number of traps containing a species was used as a surrogate for the number of colonies found. Because a single colony can field many workers, the number of workers collected is not an appropriate measure of the abundance of colonies, which is the true measure of the resilience of a social insect species to perturbation (Wilson 1962; Pamilo and Crozier 1997; Chapman and Bourke 2001).

### Calculating complementarity between transects

Complementarity is a measure of distinctness in species composition across the altitudinal transects (Gotelli and Colwell 2001). The complementarity between neighbouring transects is computed from:

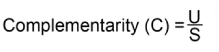

where *U* is the number of species unique to either transect and *S* is the number of species occurring at both transects.

### Deriving evolutionary distinctiveness measure

**Figure 2.**
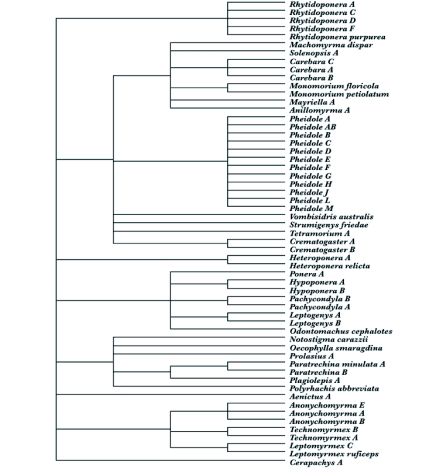
Phylogeny of the species in this study as inferred from their systematic nomenclature. The levels used were subfamily, tribe, genus, and species.

The evolutionary distinctiveness measure used in this study is evolutionary history (EH). The EH of ground dwelling ants was obtained by converting the systematic nomenclature of Bolton (2003) into an inferred phylogeny and the recording of the occurrences the ant species across the six altitudinal transects (Figure 2) using the program TREEMAKER (Crozier et al. 2005). A branch of equal length was allowed for each level in the hierarchy of the inferred phylogeny and the EH of ground dwelling ants were computed as the length of tree retained between them, always including the root of the phylogeny. Thus, two species in the same genus have a distance of 2 between them, in the same tribe but different genera one of 4, in the same subfamily but different tribes one of 6, and in different subfamilies one of 8.

### Establishing conservation priorities

Three diversity measures: estimated species richness, sites complementarity, and evolutionary history, were used in the assessments of conservation priorities using the incidences of ground dwelling ants across the altitudinal gradient in the Australian Wet Tropics. TREEMAKER enables conversion of a scheme of systematic nomenclature to an inferred phylogeny along with distributional data, and the resulting file can be read by the program MeSA to enable calculation of these biodiversity measures for each site or combination of sites (Crozier et al. 2005). Conservation assessments were achieved based on diversity preserved by conserving the set of sites with highest diversity measures. To do this the best combination of species richness, sites complementarity, and evolutionary history of the species preserved was gauged.

## Results

### Diversity of ground dwelling ants

**Figure 3.**
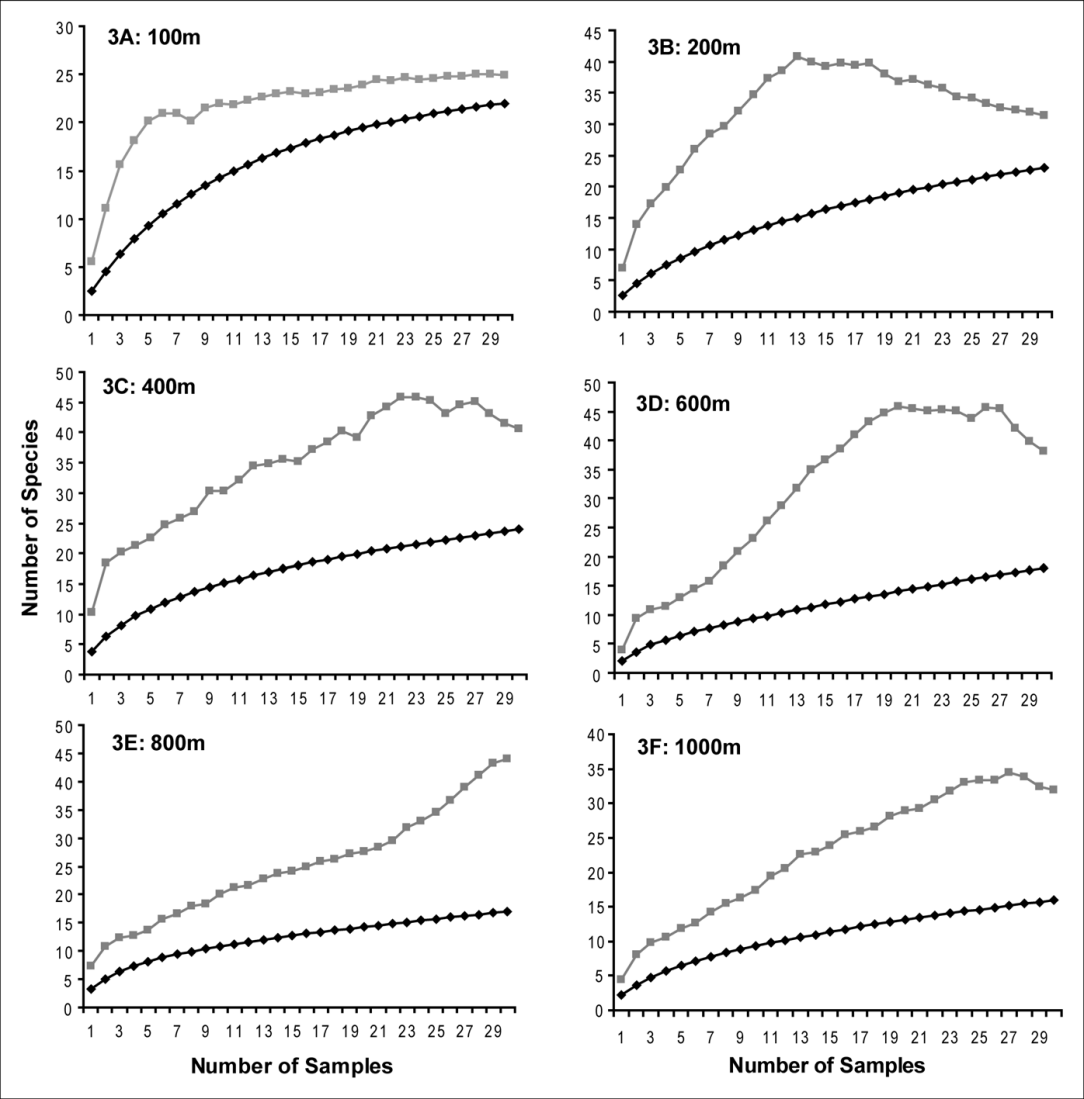
Observed (solid black line) and estimated (gray line) species richness (using the Chao2 richness estimator) of ants across six sites in an altitudinal transect (100, 200, 400, 600, 800, and 1000 m).

**Figure 4.**
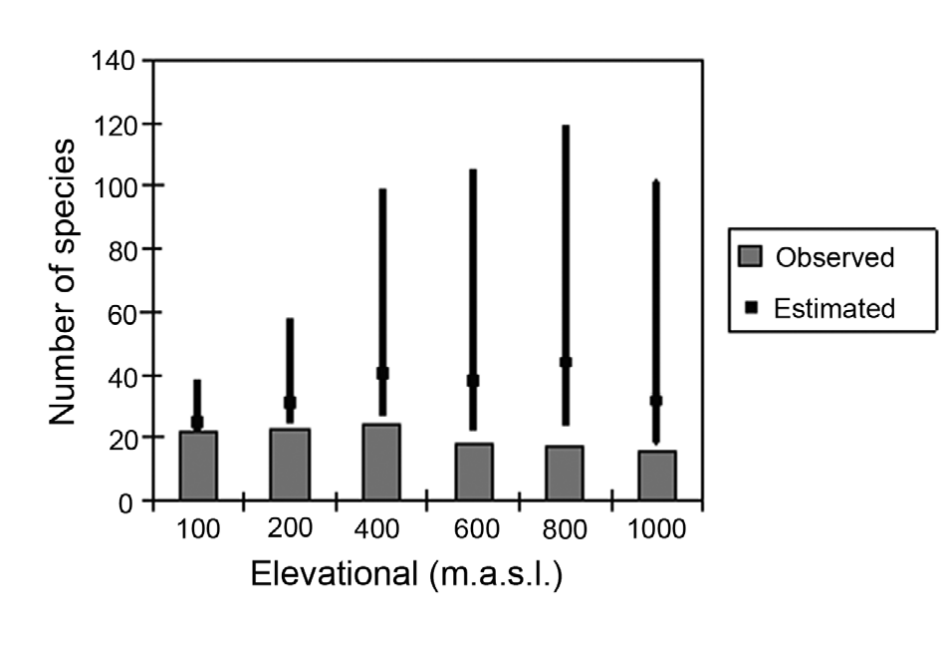
Observed and estimated species richness (Chao 2) with 95% confidence intervals.

Altogether, 56 species of ants from 34 genera and 10 sub-families were recorded from two seasons of pitfall trapping along the altitudinal gradient in the Australian Wet Tropics. The accumulation curves for altitudinal bands (Figure 3) predict an increase in expected species richness for a given level of additional sampling effort, while the diversity estimator estimate the amount of additional sampling required to reach the predicted species richness generated from species accumulation curves (Figure 4). The sampling was considered to be sufficient if the observed species richness reaches at least half the estimated richness (Chao and Lee 1992). Examining the sampling effort for this study, it appears that altitudinal sites 400, 600, 800 and 1000 m were still under-sampled for ground dwelling ant assemblages. This could mostly be due to increasing habitat complexity in these localities.

### Complementarity

The turnover in ants assemblages across altitudinal transects were computed as percentage complementarity that varies from 0 (when the assemblages are identical) to 100% (when the assemblages are completely distinct) (Table 1). The matrix of complementarity values between transects shows a moderate level of distinctness between neighboring transects (*c*. 69%). This implies that species composition of ant communities changed fairly rapidly along the altitudinal gradient.

**Table 1.**
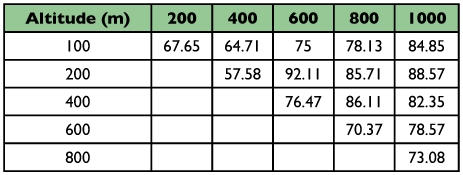
Percentage pair-wise complementarity of ant species across six altitudinal sites in the Australian Wet Tropics

### Evolutionary history

The evolutionary history preserved by each transect, in isolation, is given in Table 2.

**Table 2.**
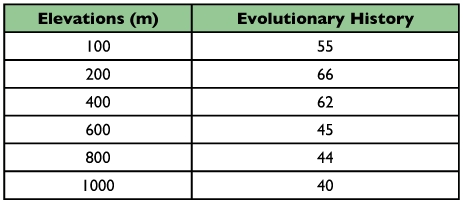
Evolutionary history value of altitudinal transects inferred from systematic nomenclature and incidences of ants

### Conservation assessments based on sites combinations

Six altitudinal sites generate altogether 62 combinations of sites. For each set of numbers of sites retained (6, 5, 4, 3, 2, or 1) the set that preserves the most biodiversity is shown in Table 3. Retaining all sites preserves 56 species with an EH of 114 and complementarity of 0.36. The best 5-sites combination is 100, 200, 400, 800, and 1000 m. This result agrees with the hypothesis that the middle elevation is the overlapping zone between two high- and low-altitude faunas. Further reducing the number of sites generates two optimum values for site combinations depending on the diversity metrices used for assessment. Omitting either the 200 or the 1000 m site will result in preservation of the same number of species (50 spp.). However, if evolutionary distinctness were used for assessment, omitting site 1000 m (i.e., site combination {1,2,4,8}) yields a higher EH value (105) than omitting the 200 m site (103). Complementarity yields a different weighting to EH, with higher complementarity at combination {1, 4, 8, 10}. The latter value implies that there are more species that are restricted to 1000 m compared to 200 m. Dropping a further site results in the site combination (100, 400, 800 m) that preserves 44 species with a value of EH of 95 and one of complementarity of 0.68 (Table 3). Lastly, the optimum choice for preservation of only two sites is 400 and 800 m, preserving 36 species with an EH value of 80 and one of 0.86 for complementarity (Table 3).

**Table 3.**
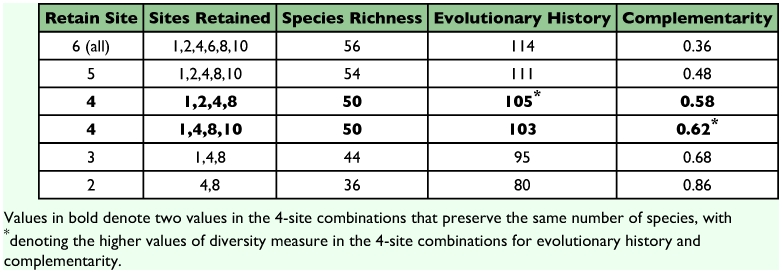
Site combinations with the highest biodiversity values

## Discussion

### Diversity of ground dwelling ants

Studies of ants species richness along altitudinal gradients have shown two patterns, a decrease in species number with altitude (Bruehl et al. 1999) or species richness peaking at intermediate elevations (Sanders 2002). In this study, species richness is fairly uniform at lower elevations (100, 200, and 400 m) with slight decrease at higher elevations (600, 800, and 1000 m). The pattern of estimated species richness is also fairly uniform across the altitudinal gradient, with lower estimated species richness at lower elevations (100 and 200 m) and higher estimates of species richness at higher elevations (400, 600, 800, and 1000 m). This interpretation is also consistent with the findings from vertebrate studies across altitudinal gradients in Australia's Wet Tropics (Williams 1997). The confidence intervals for estimated species richness at higher elevations are very wide. This indicates insufficient sampling and introduces uncertainties in the species richness data for higher elevations. Hence the pattern obtained from observed and estimated species richness in this study might not be reliable enough to indicate the underlying patterns of ant species richness across the complete altitudinal gradient. However, the wide confidence intervals of estimated species richness at higher elevations also indicate more diverse ground dwelling ant species at these elevations. The uncertainties of the pattern of ground dwelling ants could be reduced either by increasing the number of traps or incorporating additional trapping methods into the existence sampling regime at higher elevations. Therefore, further studies incorporating such changes would test our tentative conclusions of the pattern of altitudinal variation of ground dwelling ants in the Australian Wet Tropics.

### Altitudinal pattern of ground dwelling ants

The complementarity analysis revealed a narrow altitudinal range for Australian Wet Tropics ground dwelling ants (*c* 69% distinctness between altitudinal transects). As with altitudinal studies from Panama (Olson 1994) and Borneo (Bruehl et al. 1999), all communities sampled differed markedly between neighboring altitudinal sites. This pattern seems to be fairly common for tropical arthropod species (Janzen 1967). In our study, the highest composition turnover was found between the 400 and 600 m transects, which implies the presence of two ant assemblages across this altitudinal gradient. Species found from 100 to 400 m can be tentatively assigned to a low altitude assemblage and species found from 600 m upwards to a high altitude assemblage. The tentative assignment of low and high altitude ant assemblage could be influenced by site factors (Bruehl et al. 1999). The apparent undersampling of altitudinal sites 400, 600, 800, and 1000, may be mostly due to increased habitat complexity in these localities. Further, the moderate level of distinctness between transects is consistent with the species composition of ant communities changing fairly rapidly along the altitudinal gradient.

High turnover value for arthropod species along the altitudinal gradient could be attributed to the dispersal and distributional range of this group of organisms. Most altitudinal pattern studies were designed for fauna such as birds or mammals that have larger ranges and longer dispersal distances than arthropod species. Conservation managers usually draw information from those studies that serve to reveal pattern at moderate to large spatial scales. This strategy allows decisions to be made on a national level. However, the narrow altitudinal ranges of arthropod species merit concern when formulating conservation strategies for areas such as the Australian Wet Tropics. This concern arises because climate change has been identified as the biggest threat to the biodiversity of this region and the whole Wet Tropics region consists of patches of highlands that harbour most of the endemics found there. The projected upward elevational shifts of organisms due to warming indicate the high vulnerability to extinction of organisms with narrow altitudinal bands such as arthropod species and highland specifics.

### Implications of complementarity and evolutionary history on conservation assessment

Several measures of biodiversity were computed, namely species richness, complementarity and evolutionary history (using systematics as a surrogate for phylogeny). Conservation decisions should be based on choosing the most diverse site combinations based on the diversity measures available. The altitudinal pattern of ground dwelling ants in the Australian Wet Tropics conforms with a peak at middle elevations (400 m). We hypothesized that this diversity peak in mid-elevation results from overlapping low altitude and high altitude ant assemblages. Hence, if one site has to be dropped, omitting the 400 m transect retains the highest diversity site combination (Table 3). Considering all the indicators yields a relatively soundly based conservation recommendation.

Further reduction in the number of sites preserved generates two sets of conflicting site combinations depending on the diversity measures used for assessment (Table 3). The first optimum 4-sites combination {1,2,4,8} has a higher EH value. This is caused by the presence of two subfamilies (Aenictinae and Cerapachyinae) that were collected only at 200 m. There is only one genus worldwide of Aenictinae. These ants do not build stationary nests but have a nomadic lifestyle and conduct raids using large numbers of workers (Gotwald 1995). The nomadic life pattern may lead to rarity in that a large area is needed to support these mobile group hunters, and there was only one collection of this species, at 200 m. Cerapachyines are specialist predators of other ants. The low collection of this species could be due to their small colonies (Wilson 1959) and under-sampling. The second optimum 4-sites combination {1,4,8,10} yielded a higher complementarity value. Three ant species (*Carebara C*, *Mayriella A*, and *Anillomyrma A*) were collected only from 1000 m and are therefore treated as restricted to this elevation. The presence of these species at 1000 m transect is the cause of the high complementarity value for this 4-sites combination. The results of more ground dwelling ant species that are restricted to highlands is consistent with the findings from vertebrate studies across altitudinal gradients in Australian Wet Tropics (Williams 1997).

The choices for priority areas for conservation might differ depending on the diversity measures used. The straightforwardness of species richness and its estimator provide the underlying pattern for the ant assemblage in a spatial gradient. Complementarity yields an indication of the distinctness of ant assemblages between sites. Evolutionary history yields a measure of the evolutionary depth of an assemblage preserved given a particular combination of sites, without asking about the distribution of species between sites. The main aim of conservation is to preserve a set of areas that harbor the most distinct organisms from each other. Traditional conservation approaches rarely take into the account the evolutionary relationships between the organisms, hence a set of areas with high complementarity value are favored over a lower value. In this paper we provide a practicable approach of inferring a phylogeny using systematic nomenclature to derive a measure of evolutionary distinctness. This approach allows the wider scope to conservation assessment by adding this measure. Even though in this case these measures led to conflicting results yielding two optimal 4-sites combinations, both measures revealed more information about the existing pattern rather than using one diversity measure alone. We suggest that the aim in conservation assessment should be the preservation of the set of sites harboring the most distinctive organisms, effected by using a measure that involves evolutionary relationships between the organisms.

To conclude, the generations of sites combinations and simultaneous computations of various diversity measures presents a systematic approach to conservation assessment that allows a better understanding to the existing biodiversity pattern. The biodiversity preserved by conserving a set of sites can therefore be estimated by species richness, complementarity, and evolutionary history. This approach is useful to understand the diversity pattern and identify the most diverse sites combinations across an altitudinal gradient that could ensure the preservation of terrestrial ground dwelling invertebrates in the Australian Wet Tropics.

## Figures and Tables

**Table:**
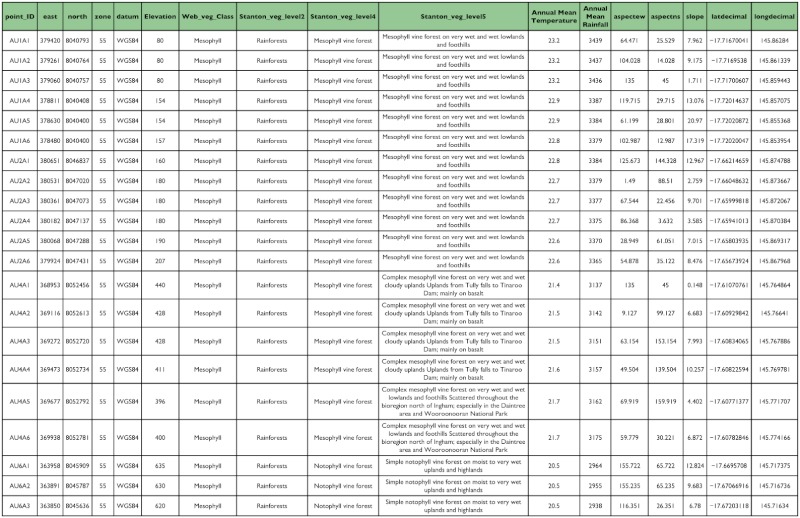
Geographical descriptions of sampling sites at Atherton Uplands.

## References

[bibr01] Agosti D, Majer JD, Alonso LE, Schultz TR (2000). *Ants: Standard methods for measuring and monitoring biodiversity*..

[bibr02] Andersen AN, Fisher A, Hoffmann BD (2004). Use of terrestrial invertebrates for biodiversity monitoring in Australian rangelands, with particular reference to ants.. *Austral Ecology*.

[bibr03] Bolton B (2003). Synopsis and classification of Formicidae.. *Memoirs of the American Entomological Institute*.

[bibr04] Bruehl CA, Mohamed M, Linsenmair KE (1999). Altitudinal distribution of leaf litter ants along a transect in primary forests on Mount Kinabalu, Sabah, Malaysia.. *Journal of Tropical Ecology*.

[bibr05] Chao A (1987). Estimating the population size for capture-recapture data with unequal catchability.. *Biometrics*.

[bibr06] Chao A, Lee S-M (1992). Estimating the number of classes via sample coverage.. *Joumalof the American Statistical Association*.

[bibr07] Chapman RE, Bourke AFG (2001). The influence of sociality on the conservation biology of social insects.. *Ecology Letters*.

[bibr08] Colwell RK (2005). EstimateS: statistical estimation of species richness and shared species from samples. Version 7.5.. *User's guide and application.*.

[bibr09] Cowling RM, Pressey RL (2001). Rapid plant diversification: planning for an evolutionary future.. *Proceedings of the National Academy of Sciences of the United States of America*.

[bibr10] Crozier RH (1992). Genetic diversity and the agony of choice.. *Biological Conservation*.

[bibr11] Crozier RH (1997). Preserving the information content of species: genetic diversity, phylogeny, and conservation worth.. *Annual Review of Ecology and Systematics*.

[bibr12] Crozier RH, Dunnett LJ, Agapow P-M (2005). Phylogenetic biodiversity assessment based on systematic nomenclature.. *Evolutionary Bioinformatics Online*.

[bibr13] Faith DP (1992). Conservation evaluation and phylogenetic diversity.. *Biological Conservation*.

[bibr14] Forest F, Grenyer R, Rouget M, Davies TJ, Cowling RM, Faith DP, Balmford A, Manning JC, Proches S, van der Bank M (2007). Preserving the evolutionary potential of floras in biodiversity hotspots.. *Nature*.

[bibr15] Frith C (1992). *Australia's wet tropics life, including the Daintree region*..

[bibr16] Gotelli NJ, Colwell RK (2001). Quantifying biodiversity: procedures and pitfalls in the measurement and comparison of species richness.. *Ecology Letters*.

[bibr17] Gotwald WH (1995). *Army ants: the biology of social predation*..

[bibr18] Hilbert DW, Ostendorf B, Hopkins MS (2001). Sensitivity of tropical forests to climate change in the humid tropics of north Queensland.. *Austral Ecology*.

[bibr19] Hoffmann BD, Andersen AN (2003). Responses of ants to disturbance in Australia, with particular reference to functional groups.. *Austral Ecology*.

[bibr20] Janzen DH (1967). Why mountain passes are higher in the tropics?. *The American Naturalist*.

[bibr21] Kaspari M, Majer JD, Agosti D, Majer JD, Alonso E (2000). *Using ants to monitor environmental change*..

[bibr22] Lawton JH, Bignell DE, Bolton B (1998). Biodiversity inventories, indicator taxa and effects of habitat modification in tropical forest.. *Nature*.

[bibr23] May RM (1994). Conceptual aspects of the quantification of the extent of biological diversity.. *Philosophical Transactions of the Royal Society B: Biological Sciences*.

[bibr24] Nee S, May RM (1997). Extinction and the loss of evolutionary history.. *Science*.

[bibr25] Nix HA, Switzer MA (1991). *Rainforest animals: Atlas of vertebrates endemic to Australia's wet tropics*..

[bibr26] Olson DM (1994). The distribution of leaf litter invertebrates along a neotropical altitudinal gradient.. *Journal of Tropical Ecology*.

[bibr27] Pamilo P, Crozier RH (1997). Population biology of social insect conservation.. *Memoirs of Museum Victoria*.

[bibr28] Sanders NJ (2002). Elevational gradients in ant species richness: area, geometry, and Rapoport's rule.. *Ecography*.

[bibr29] Shattuck SO (2000). *Australian ants: their biology and identification*..

[bibr30] Singh G., Smith JMB (1982). *Environmental upheaval: Vegetation of Australia during the quaternary*..

[bibr31] Smith MA, Fisher BL, Hebert PDN (2005). DNA barcoding for effective biodiversity assessment of a hyperdiverse arthropod group: the ants of Madagascar.. *Philosophical Transactions of the Royal Society B: Biological Sciences*.

[bibr32] Vane-Wright RI, Humphries CJ, Williams PH (1991). What to protect? Systematics and the agony of choice.. *Biological Conservation*.

[bibr33] Walsh PD, Henschel P, Abernethy KA (2004). Logging speeds little red fire ant invasion of Africa.. *Biotropica*.

[bibr34] Weitzman ML (1992). On diversity.. *The Quarterly Journal of Economics*.

[bibr35] Williams SE (1997). Patterns of mammalian species richness in the Australian tropical rainforests: are extinctions during historical contractions of the rainforest the primary determinants of current regional patterns in biodiversity?. *Wildlife Restoration*.

[bibr36] Williams SE, Bolitho EE, Fox S (2003). Climate change in Australian tropical rainforests: An impending environmental catastrophe. Proceedings of the Royal Society of London.. *Series B, Biological sciences*.

[bibr37] Wilson EO (1959). Some ecological characteristics of ants in New Guinea rainforests.. *Ecology*.

[bibr38] Wilson EO (1962). The Trinidad cave ant Erebomyrma (=Spelaeomyrmex) urichi (Wheeler), with a comment on cavernicolous ants in general.. *Psyche*.

[bibr39] Wiltshire K (1986). *Tropical rainforests of North Queensland: Their conservation significance*..

